# An investigation of the long-range and local structure of sub-stoichiometric zirconium carbide sintered at different temperatures

**DOI:** 10.1038/s41598-020-59698-6

**Published:** 2020-02-20

**Authors:** Dhan-sham B. K. Rana, Eugenio Zapatas Solvas, William E. Lee, Ian Farnan

**Affiliations:** 1 Department of Earth Sciences, University of Cambridge, Downing Street, Cambridge, CB2 3EQ UK; 2 Centre for Nuclear Engineering, Department of Materials, Imperial College London, London, SW7 2AZ UK

**Keywords:** Ceramics, Mechanical properties, Materials science

## Abstract

ZrC_1−x_ (sub-stoichiometric zirconium carbide), a group IV transition metal carbide, is being considered for various high temperature applications. Departure from stoichiometry changes the thermo-physical response of the material. Reported thermo-physical properties exhibit, in some cases, a degree of scatter with one likely contributor to this being the uncertainty in the C/Zr ratio of the samples produced. Conventional, methods for assigning C/Zr to samples are determined either by nominal stochiometric ratios or combustion carbon analysis. In this study, a range of stoichiometries of hot-pressed ZrC_1−x_ were examined by SEM, XRD, Raman spectroscopy and static ^13^C NMR spectroscopy and used as a basis to correct the C/Zr. Graphite, amorphous, and ZrC_1−x_ carbon signatures are observed in the ^13^C NMR spectra of samples and are determined to vary in intensity with sintering temperature and stoichiometry. In this study a method is outlined to quantify the stoichiometry of ZrC_1−x_ and free carbon phases, providing an improvement over the sole use and reliance of widely adopted bulk carbon combustion analysis. We report significantly lower C/Zr values determined by ^13^C NMR analysis compared with carbon analyser and nominal methods. Furthermore, the location of carbon disassociated from the ZrC_1−x_ structure is analysed using SEM and Raman spectroscopy.

## Introduction

ZrC_1*−x*_ (sub-stoichiometric zirconium carbide), is under consideration for its use in generation IV nuclear fuel coatings due to its favourable mechanical, thermal, neutronic, and fission product retention properties^1–4^. This combination of characteristics is derived from the combination of its metallic electronic properties and its ceramic properties^5^.

ZrC crystallises in the rocksalt structure with carbon atoms located in the octahedral interstitial sites. When carbon is removed from the ZrC structure, significant changes are seen in the physical and thermal properties. Scatter in reported data exists in the variation of the physical properties with the atomic C/Zr ratio. The scatter in the data potentially arises due to the combination of two factors: inaccurate composition measurements (C/Zr ratio) resulting in misreferenced physical properties; and the contribution of interstitial impurities such as oxygen and nitrogen leading to a range values for several themo-physical properties.

Non-monotonic trends in material properties have been observed for physical properties, such as the lattice parameter. Assuming the sub-stochiometric material is comprised exclusively of ZrC and no contaminant species the configuration ordering of carbon atoms as the concentration of vacancies increases may be a contributing factor to the occurrence of these non-monotonic trends. As understanding of how these vacancies interact and how this affects material properties is crucial to evaluating the suitability of ZrC_1*−x*_ for nuclear materials. Fundamentally, correct determination of the carbon content of sub-stoichiometric ZrC_1*−x*_ is vital in order to accurately reference the properties of ZrC_1*−x*_ to its stoichiometry.

Numerous fabrication routes exist for producing solid ZrC_1*−x*_ samples^6–11^. However, reactive hot-pressing is commonly used as a technique for creating samples near theoretical density^12–15^. The vast majority of experimental studies quote nominal^12,16–18^ and combustion carbon analysis^6,19–24^ values for carbon content, with this being used as a reference point for the attribution of material thermo-mechanical property values. Little previous work has been undertaken to evaluate the accuracy of standard carbon content characterisation techniques. This study proposes alternative characterisation methods that can provide quantitative corrections and examines how distributions of carbon atoms vary with sintering temperature and stoichiometry and explores the accuracy of commonly used carbon analysis techniques using static solid state ^13^C NMR (nuclear magnetic resonance).

## Experimental Methods

### Fabrication method

Samples with nominal stoichiometries were fabricated under a vacuum atmosphere by reactively hot-pressing (FCT Systeme GmbH, Germany) mixtures of precursor powders; *ZrH*_2_ (453330317, 2.4 μm, Rockwood Lithium GmbH, Germany) and graphite powder (282863, <20 μm, synthetic, Sigma-Aldrich, USA) according to the reaction (1) below.

1 $$Zr{H}_{2}+C\,\to ZrC+{H}_{2}$$

The reactant powders were mixed to the desired stoichiometric ratio under inert argon gas glove-box conditions. These powders were then dry milled in nylon jars with ZrO_2_ balls (~1.0 cm in diameter) in a Retsch PM100 planetary miller (Resch, Han, Germany) for; 30 minutes at 150 rpm with milling direction reversal occurring for 5 minute periods in order to produce homogenous mixtures.

The milled base powders (see Supplementary Table T1) were loaded into graphite dies (3.5 cm diameter) and separated from the die surface and each other by layers of commercial grade *grafoil (Erodex (UK) Ltd, UK) (0.05 cm in thickness)*. They were heated at a rate of 10 °C/min to 1400 °C with a contact load of 5 kN; held at 1400 °C for 60 minutes at a contact load of 21.3 kN; heated to the desired sintering temperatures (2000 °C, 1700 °C, 1500 °C) at a rate of 10 °C/min under a contact load of 30 kN and held at these temperatures for 1 hour. The contact load was then reduced to 5 kN, the heaters were turned off allowing the samples to cool to ambient temperature. The nominal stoichiometries produced are listed in Table 1.

**Table 1:** Nominal and carbon analysed values for ZrC_1*−*x_ samples with the error on the carbon analyser calculated from the standard deviation of triplicate measurements.

Sintering temperature (°C)	Nominal C/Zr	analysed carbon content ± error
2000	1.00	1.00 ± 0.01
	0.80	0.85 ± 0.00
	0.60	0.64 ± 0.00
1700	1.00	0.96 ± 0.00
	0.80	0.73 ± 0.01
	0.60	0.64 ± 0.00
1500	0.95	0.95 ± 0.01
	0.70	0.73 ± 0.01
	0.65	0.64 ± 0.00

Post-sintering, approximately 0.25 cm of each sample pellet was removed radially and axially (both faces) of the pellet with a circular grinder. This ensured, that any excess, graphite that could affect carbon measurements was removed. The pellets were cleaned using high purity ethanol. Blocks measuring 0.5 cm × 0.5 cm and 200 μm in thickness were cut from the sintered pellets using a wire electrical discharge machine. In order to examine the microstructure using SEM (scanning electron microscopy), the samples sintered at 2000 were polished to a mirror finish using diamond polishing paste of decreasing particulate size with the final step being 0.2 μm.

A proportion of each dense pellet was reduced to a fine powder by hand. The pellet was broken up using a stainless-steel punch and hammer on a clean foil coated anvil and the particle-size was reduced using an agate mortar and pestle. The semi-metallic nature of ZrC_1*−*x_; means it possesses a skin depth that attenuates the RF (radio frequencies) used in NMR (nuclear magnetic resonance) spectroscopy. Thus, it was necessary to reduce the particle diameter below the skin depth to allow for total signal penetration. A skin depth of ~34 µm was calculated from correlations in the literature^1,2,25^. To achieve this diameter, the powdered samples were further dry milled, sieved to less than ~25 µm. The powdered samples were further screened for any magnetic particles that may have arisen from the preparation apparatus using a very high magnetic field.

### Carbon analysis

The total carbon content of the sample was measured using a HORIBA EMIA-320 V2 Carbon and Sulphur chemical analyser. The carbon analyser uses a furnace to combust the samples; a process that results in carbon dioxide gas being emitted from the sample. The amount of carbon dioxide produced is measured by calibrated infrared detectors yielding the total carbon content of the sample.

Oxygen and nitrogen were used as the carrier and operating gases in the analyser, respectively. Aluminium oxide (50–70%) and silica (30–50%) ceramic crucibles, previously heated to 1000 °C for 30 minutes in a muffle furnace to remove any carbon impurities and then stored in a desiccator, were used to contain the samples in the analyser furnace chamber. Prior to running samples, the analyser was calibrated. Quantification of carbon present in a batch of crucibles and the accelerant powers (copper and iron pellets purchased from HORIBA - used to elevate combustion temperatures) was undertaken by running three crucibles and three crucibles containing accelerant. Powdered tungsten carbide standard of known total carbon content (HORIBA 30 mg-BSC–CRM No. 352/1 from The Bureau of Analysed Samples, UK) was used to calibrate the analyser. For a given sample run including the tungsten calibration run an empty crucible was removed from the desiccator using tongs and placed on a Mettler Toledo measurement scale connected to a computer. A small amount of powdered sample (~1.00 g) was placed in the crucible using a spatula, the sample mass was recorded in the HORIBA analyser software. The sample was placed in the furnace and the analyser routine was activated–outputting mass percent of the carbon in the analyser. Triplicate measurements were undertaken for each sample to provide a basis for error quantification.

### ^13^C nuclear magnetic resonance spectroscopy

Static, room temperature, ^13^C NMR was undertaken on all samples to investigate carbon local environments in the sample, providing the basis for distinguishing different carbon sites and quantitatively determine their relative abundance. Static NMR was used as spinning semi-metallic samples, such as ZrC, is difficult due to the opposing Lorentz force generated by turning a conductor in a magnetic field. In addition, preliminary spinning experiments at 20 kHz in a 3.2 mm rotor showed no spectral narrowing compared with a static sample–hence, static NMR was used for all experiments in this study. Static room temperature ^13^C NMR spectroscopy was performed on a Varian Infinity spectrometer operating at a frequency of 100.603 MHz, for ^13^C with a 9.39 T magnet. To minimise signal contributions from NMR rotor components, the crushed samples were loaded into 7.5 mm zirconia rotors with aluminium nitride spacers (replacing standard carbon containing polymeric spacers). The Hahn echo technique was employed to obtain spectra, using the sequence: π/2 (4.70 μs), delay (30.00 μs), π (9.40 μs), each acquisition was separated by a pulse delay (9.00 s), which was determined to be the saturating condition by variable delay experiments. Around 10,000 echoes were averaged, and Fourier transformed from the echo top; with a Lorentzian line broadening of 200 Hz being applied before transformation. The precursor graphite powder spectrum was collected using a single pulse sequence with a pulse delay of 0.10 s. All NMR spectra were referenced to tetramethylsilane (TMS), via a secondary reference to solid adamantane (38.52 ppm^26^) which was acquired using a single π/2 pulse followed by a pulse delay (1.00 s).

As the carbon nuclei are being directly observed via NMR, the integral of a peak in the spectrum corresponds to the number of carbon atoms present in that unique chemical environment. The analysis of the contribution of different carbon atoms to each sample spectrum was carried out using a non-linear least square fitting routine (IgorPro) with Gaussian, Lorentzian and Voigt peak profiles. The proportion of carbon atoms in ZrC_1*−*x_ sites was found by taking the ratio of the peak value with respect to the total peak area. This can be used to correct the value of the carbon content of the ZrC_1*−x*_ by assigning the spectral fractional integral of the ZrC_1*−x*_ peak as the corresponding fraction of the total carbon content given by the carbon analyser.

### XRD (x-ray diffraction)

Powder X-ray diffraction was conducted on a Bruker D8 Advance diffractometer in a Bragg-Brentano configuration using Cu Kα1 radiation. A standard reference of silicon was mixed into the powdered sample. A single crystal silicon zero-background sample holder was used and the X-ray diffractogram was collected from 20° to 105° 2θ at room temperature with the – step interval being 0.015°. XRD was used to confirm the rock-salt structure^1,2,17,27^ of ZrC_1*−x*_, to determine if any other phases were present in the sample^15,28,29^ and, using Rietveld refinement in the TOPAZ academic software suite, to determine the lattice parameter and the associated error.

### SEM

SEM was conducted on the 2000 °C samples to investigate the microstructure and more specifically how the carbon content affects the grain size. A Quanta-6F0F SEM was used to collect BSE and SE (backscattered and secondary electron) images using Everhart Thornley and Circular Backscatter Detectors, respectively. Solid pellet samples were mounted onto aluminium stubs using double sided carbon tape. Samples were imaged at a working distance of 13 mm using SE and BSE detectors with an accelerating voltage of 5 kV in high vacuum. Chemical analysis of different regions in the images were probed by energy dispersive x-ray spectroscopy (EDX) using a Bruker 6130 XFlash and analysed using the ESPIRIT software. EBSD maps were collected on the sample to quantify the grain size, using an accelerating voltage of 25 kV. EBSD setup *stub containing the sample was mounted onto a stage orientated at 70°.

### Raman spectroscopy

Raman spectroscopy was conducted on solid ZrC_1*−*x_ samples to investigate the presence of different allotropes of carbon in the sample and their morphology^30^. Sections of the sintered pellets, 0.5 cm × 0.5 cm, were introduced into a Horiba Jobin Yvon confocal LabRam300 Raman spectrometer with green laser wavelength of 532 nm at 90 mW. An x50 Olympus objective lens with 1000 µm confocal aperture and a holographic grating of 1800 slits per mm was used and the spectrometer was calibrated using a silicon wafer which has a characteristic Raman line at 520 cm^−1^. Raman spectra were obtained from different regions of the sample b adjusting the location of the focal point of the spectrometer with an in-axis optical microscope.

## Results

### Carbon analyser results

Table 1 presents the results of total carbon analysis of sintered ZrC_1*−*x_ pellets and the nominal content defined by the stoichiometry of the initial mixed powders. Carbon analysis values correspond to the total carbon content determined by the carbon analyser.

Most samples show a higher analysed carbon content as compared with nominal values, with a third of the samples showing a lower analysed carbon–two of these samples belong to the set of samples sintered at 1700 °C. The total carbon content of the lower stoichiometries across all sintering temperatures were shown to have similar values. The total carbon content the samples sintered at 1500 °C and 1700 °C being above and below nominal stoichiometry. Samples sintered at 1700 °C and 1500 °C also show a larger deviation from nominal stoichiometry at intermediate stoichiometries.

### ^13^C NMR

**Figure 1:**
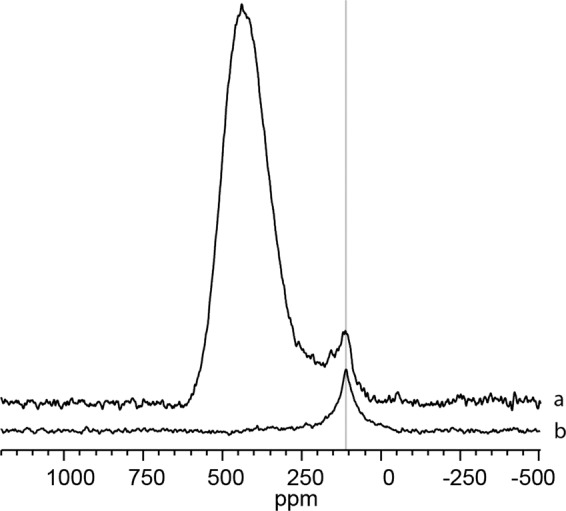
(**a**) ^13^C NMR spectrum of ZrC_1*−*x_, nominal C/Zr = 0.60, sintered at 2000 °C (**b**) spectrum of precursor graphite located at ~113 ppm.

Figure 1 (a) shows the ^13^C NMR spectrum of nominal C/Zr = 0.60 sintered at 2000 °C offset, and plotted on the same axis, Fig. 1(b) shows the spectrum of the graphite precursor powder.

The location of the peak arising from graphite is located ~113 ppm^31^, its presence can be seen in a typical ZrC_1*−*x_ sample spectrum clearly showing the presence of graphite within the sample.

**Figure 2:**
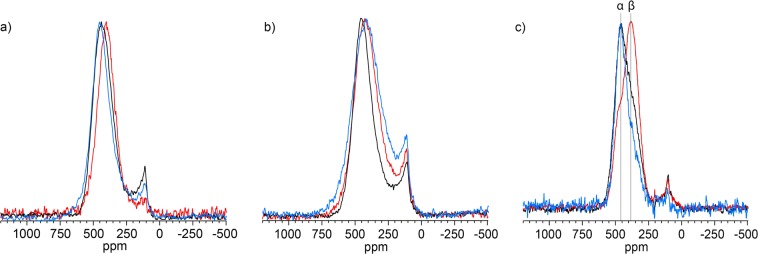
^13^C NMR spectra with normalised peak intensities for different stoichiometries sintered at (**a**) 2000 °C, (**b**) 1700 °C, (**c**) 1500 °C. Red, black and blue spectrum traces correspond to nominal C/Zr = 1.00, 0.80, 0.60 for 2000 °C and 1700 °C - C/Zr = 0.95, 0.70, 0.65 for 1500 °C; α and β denote resolved peaks in the 1500 °C spectra.

**Figure 3:**
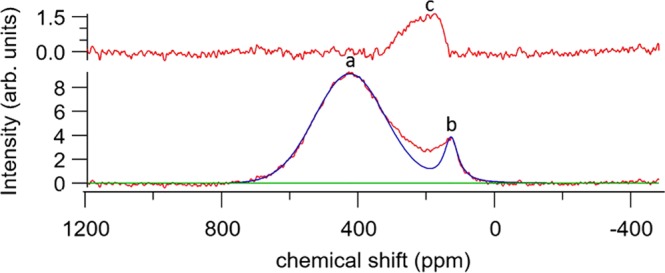
Deconvolution of the ^13^C NMR peaks in a sample sintered at 1700 °C with nominal C/Zr 0.80. The bottom spectrum shows the Gaussian peak fitted to the ZrC_1*−*x_ carbon resonance at 419ppm (**a**) and a Voigt peak at 113 ppm to the resonance of graphite carbons (**b**). The top profile shows the residual when (**a**) and (**b**) are subtracted from the original line shape, revealing an additional resonance labelled (**c**).

Figure 2 shows the static ^13^C NMR spectra for all samples fabricated grouped by sintering temperature and plotted on a normalised scale. The ZrC_1*−*x_ resonance peak of the samples sintered at 2000 °C (Fig. 2a) shifts from higher to lower ppm with increasing carbon content. The CG (peak centre of gravity) of the ZrC_1*−x*_ peak varies from 434 to 404 ppm corresponding to a peak shift of ~30 ppm. The 1700 °C sintered samples (Fig. 2b) show a contrasting trend to the 2000 °C sintered samples with the centre of gravity of the peak varying from the lower to higher ppm with increasing carbon content. Quantitatively, this corresponds to a ZrC_1*−x*_ peak shift change of ~23 ppm varying from 411 ppm to 434 ppm. The samples sintered at 1500 °C (Fig. 2c) indicate that the main ZrC_1*−x*_ peak present in nominal C/Zr = 0.65 and 0.70 samples, labelled α, appears to evolve into the peak, labelled, β as carbon content increases to a nominal C/Zr = 0.95, which also appears as a shoulder in the spectra of the nominal 0.70 and 0.65 signals. For the 1500 °C samples, the intensity of the graphite-like signal increases with increasing carbon content (Table 2). In addition to the ‘sharp’ peak at 113 ppm there is also evidence for an additional peak at ~120–300 ppm, and therefore an additional carbon local environment in these ^13^C NMR spectra, especially in the samples sintered at 1700 °C. Figure 3 (bottom) shows a representative ^13^C NMR spectrum for samples of ZrC_1*−x*_ the blue tracer line indicates the peak fits based on symmetric ZrC_1*−*x_ and graphite resonances.

**Table 2:** Compilation of the analysed and NMR corrected carbon content errors on the NMR fit are propagated through from errors on the peak fits and the error (±1σ) on the carbon analyser is taken from the triplicate measurements.

Sintering temperature (°C)	Nominal C/Zr	Analysed C/Zr ± error	Corrected C/Zr from NMR ± error	Corrected Graphite content from NMR ± error	Corrected broad carbon content from NMR ± error
2000	1.00	1.00 ± 0.01	0.95 ± 0.01	0.03 ± 0.01	0.03 ± 0.03
	0.80	0.85 ± 0.00^*^	0.75 ± 0.01	0.03 ± 0.01	0.07 ± 0.01
	0.60	0.64 ± 0.00^*^	0.50 ± 0.00^*^	0.04 ± 0.00^*^	0.09 ± 0.01
1700	1.00	0.96 ± 0.00^*^	0.81 ± 0.00^*^	0.07 ± 0.00^*^	0.17 ± 0.03
	0.80	0.73 ± 0.01	0.56 ± 0.01	0.09 ± 0.01	0.03 ± 0.01
	0.60	0.64 ± 0.00^*^	0.50 ± 0.00^*^	0.05 ± 0.00^*^	0.08 ± 0.01
1500	0.90	0.95 ± 0.01	0.88 ± 0.02	0.06 ± 0.02	0.01 ± 0.11
	0.70	0.73 ± 0.01	0.69 ± 0.01	0.03 ± 0.01	0.01 ± 0.22
	0.65	0.64 ± 0.00^*^	0.60 ± 0.01	0.00^*^ ± 0.00^*^	0.04 ± 0.13

A Voigt peak was fitted to the graphite environment as this gave the best fit. The fit was optimised using non-linear regression analysis which used the Levenberg-Marquardt algorithm. The Voigt peak parameter values (position, width, and Gaussian-Lorentzian fraction) determined from the graphite precursor were used to restrict the graphite peak fitting to its intensity. For the ZrC_1*−x*_ resonance, the peak was fitted with asymmetric Gaussian using the shape of the high-frequency (low field) side of the peak (including ‘dummy’ Gaussians to represent the missing intensity). The additional resonance observed in the residual spectrum (labelled c) located between ~120–300 ppm is difficult to fit without constraints; as its broad structure means that any fitted peak(s) infringe on the ZrC_1*−x*_ and the graphite resonances. The contribution of the broad peak to the total carbon content can be therefore quantified by subtracting the sum of the ZrC_1*−x*_ and graphite signal integrals from the total resonance signal integral. Table 2 lists C/Zr values given by the carbon analyser method, and correct values determined from the peak fitting.

### SEM analysis

**Figure 4:**
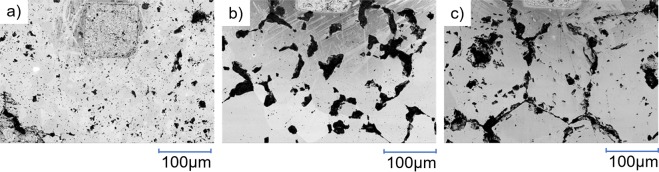
BSE images of sample sintered at 2000 °C with nominal C/Zr = (**a**) 1.00, (**b**) 0.80, (**c**) 0.60 Square markings are reference points.

Figure 4 shows low magnification BSE images taken of samples pf samples with different carbon contents sintered at 2000 °C. Sample surfaces reveal the presence of surface depressions appearing as crack like network structures that are present in all samples.

For nominal C/Zr = 1.00 the microstructure showed a significant amount of intra and intergranular features, with the latter appearing larger in size than the former. The micrographs of the nominal C/Zr = 0.80 show a preference for intergranular features which partially occupy elongated portions along the grain boundaries (Fig. 4). Samples with nominal C/Zr = 0.60 show a fully developed network of intergranular structures which distinctly trace out the grain boundaries as well as large intra granular structures. The average grain size distribution was analysed using the MTEX^32^ package in MATLAB and was found to be 16, 217 and 235 μm for nominal C/Zr 1.00, 0.80 and 0.60 respectively. A decrease in the average grain size is observed as the carbon content of the sample is increased^33^.

**Figure 5:**
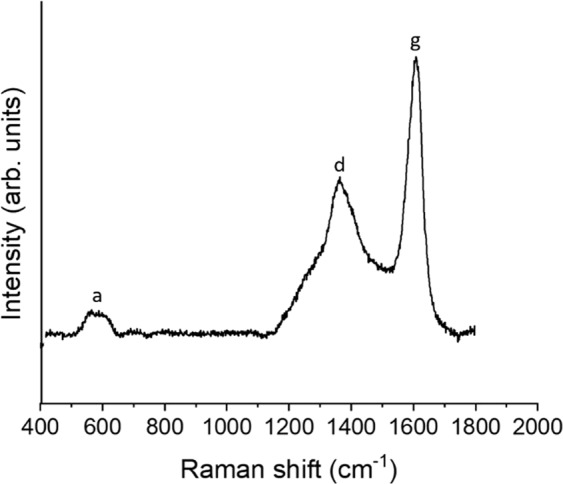
Baselined Raman spectrum sample sintered at 2000 °C for nominal C/Zr = 0.60 for intra and intergranular crack regions – the amorphous band is labelled a, the D band is labelled d, the G band is labelled g.

Figure 5 shows baseline corrected Raman spectrum of nominal C/Zr = 0.60 sintered at 2000 °C in which active modes can been seen. Two of these peaks can been attributed to sp^2^ carbon ~1359 cm^−1^ and ~1600 cm^−1^ termed the D and G peak^34–38^. The activation of D breathing mode occurs as a result of the disruption in a perfect graphite structure and its presence is indicative of disorder. The G mode can be attributed C-C stretching mode of the graphite structure^35,37,39,40^. Finally, a broad peak is present centred ~500 cm^−1^ and this has been attributed previously to amorphous carbon^30,41^.

## Discussion

^13^C NMR spectra reveal that a diverse range of unique carbon chemical environments or phases are present in all samples. One such environment that was observed was the sharp peak (~113 ppm) which is dissociated from the main ZrC_1*−x*_ peak – its structure and presence is validated by the ^13^C NMR spectra of the graphite precursor powder. This implies that extra carbon phases are present in the sample that do not exist in the ZrC_1*−x*_ structure. According to the phase diagram the homogeneous region of ZrC_1*−x*_ is located between 0.55 > C/Zr > 1.00^1,2,4^ with no extra phases present. As discussed in the methods section, a *grafoil* liner was used to line the dies utilised in the hot press. Although every effort was made to remove any bonded *grafoil* from the sample and a considerable proportion of the sample was removed from the outer faces to further limit this – there is a possibility that elevated temperatures and the presence of a carbon concentration gradient may have led to an ingress via diffusion of carbon from *grafoil* to the sample.

The presence of a clear resonance for ZrC_1*−*x_ and graphite environments are observed in the NMR spectra. However, in almost all NMR spectra this is accompanied by an additional, separate broad peak of unknown origin. It is possible that anisotropy may accompany the resonance of a carbon atom that is associated with one or more carbon vacancy in the 2nd neighbour sites. If the anisotropy were located at a low frequency, the broad carbon peak could be associated with an anisotropy of the ZrC_1*−x*_ peak. Theoretically, a stochiometric C/Zr = 1.00 structure will not exhibit anisotropy because the carbon sites in a symmetric cubic rocksalt structure have a high point symmetry. In contrast, a structure with vacancies can have a distribution of unique chemical environments and different orientations of crystallites with respect to the spectrometer magnetic field. The implication is that either there is some anisotropy associated with the static ZrC_1*−x*_ spectrum or an additional carbon environment is present in the sample.

**Figure 6:**
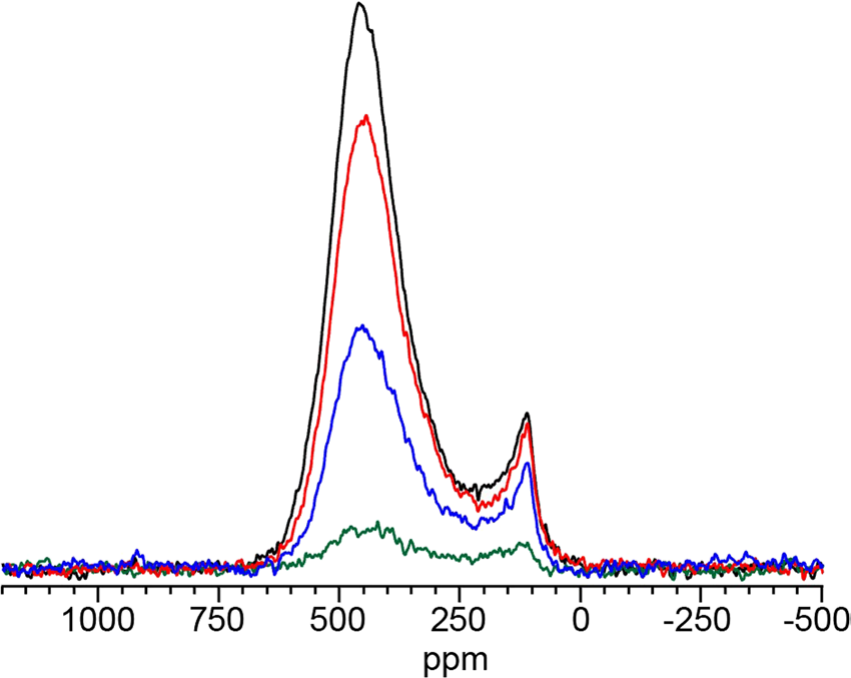
^13^C NMR the evolution of the broad structure (indicated by grey line) with respect to the pulse delay for the nominal C/Zr = 1.00 sample sintered at 1700 °C. Red, black, blue and green spectral traces correspond to pulse delays of 9.00, 3.00, 1.00 and 0.10 seconds respectively.

Figure 6 shows the relative NMR spin-lattice relaxation behaviour of the ZrC_1*−*x_, the graphite and the amorphous carbon peaks. The pulse delay for maximum signal (equilibrium signal) was found to be 9.0 s while the maximum signal for the graphite-like peak pulse delay was found to be ~3.0 s in the sample corresponding to a T_1_s of 1.8 and 0.6 s for the different type of carbons, respectively. The ZrC_1*−*x_ relaxes on a relatively long timescale whereas the saturation relaxation timescale of the broad carbon environment is more rapid. This implies that carbon atoms in the amorphous carbon exist in an environment that is chemically closer to graphite than the ZrC_1*−x*_ environment. The associated relaxation behaviour also confirms that unreacted graphite, which has a very short T_1_, is not present in the sample. The location of the broad peak indicates that this signal is due to sp^2^ type carbon and is similar to that exhibited by black fullerenes and amorphous carbon structures^34,35,39,40,42^. Comparison of the nominal carbon content and the carbon analyser data with respect to the NMR curve fitting, reveals that the carbon content cannot be accurately determined by the first two methods.

Computational investigations of vacancy ordering in ZrC_1*−*x_, by Zhang *et al*.^43^ and Xie *et al*.^44^ identified several stable stoichiometries. Comparison of these stable stoichiometries with NMR corrected C/Zr ratios shows the coincidence of the compositions of a number of these stable phases present in the samples in this study. These are highlighted in green in Table 3.

**Table 3:** Comparison of static ^13^C NMR evaluated NMR C/Zr as compared with stable Z_y_C_z_ phases computed by Zhang *et al*. (^**†**^)^43^ Xie *et al*. (^**‡**^)^44^. Errors too small to show denoted by *.

Experimentally determined			Computationally determined Stoichiometry
Sintering temperature (°C)	Nominal C/Zr	Corrected C/Zr from NMR ± error	
2000	1.00	0.95 ± 0.01	Na
	0.80	0.75 ± 0.01	Zr_4_C_3_^†^
	0.60	0.50 ± 0.00^*^	Zr_2_C_1_^†‡^
1700	1.00	0.81 ± 0.00^*^	Zr_32_C_28_^†^
	0.80	0.56 ± 0.01	Zr_32_C_18_^†^
	0.60	0.50 ± 0.00^*^	Zr_2_C_1_^† ‡^
1500	0.95	0.88 ± 0.02	Zr_8_C_7_^†^
	0.70	0.69 ± 0.01	na
	0.65	0.60 ± 0.01	na

**Figure 7:**
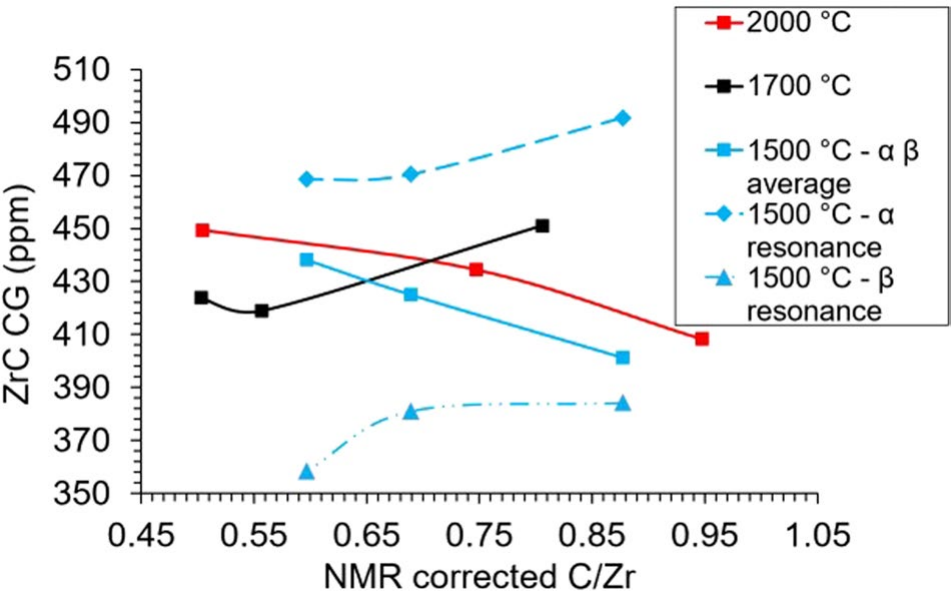
CG as determined from NMR spectrum peak fitting plotted with respect to NMR determined C/Zr for samples sintered at 2000 °C, 1700 °C and 1500 °C.

The presence of at least two unique carbon environments within the ZrC_1*−*x_ resolved in the resonances of the samples sintered at 1500 °C (Fig. 2(c)) show an interesting feature which was not seen in the other samples. Figure 7 shows the results of two Gaussian functions fitted to the 1500 °C spectral resonances whose CGs are α at ~460 ppm and β at ~370 ppm. The CG of the Gaussian functions fitted to the α and β resonances appear be the maximum and minimum positions, respectively, bounding the CG of all fabricated samples.

The location of the CGs of all samples were seen to decrease with increasing carbon content with the exception of nominal C/Zr = 1.00 sintered at 1700 °C. Overall the CG position was also seen to decrease with lower fabrication sintering temperatures apart from the 1500 °C samples due the inhomogeneity in the line shape.

For the 1500 °C samples the CG and the percentage of carbon in α and β resonances were observed to be dependent on the concentration of carbon in the sample. The percentage of carbons present in the ZrC_1*−x*_ resonance for nominal C/Zr = 0.95, 0.70, 0.65 samples were respectively for the α resonance 16%, 49%, 72% and for the β resonance 84%, 51%, 28%. The systematic evolution in occupancy of the α β carbon resonance environments varies solely as a function of the carbon content. The number of carbon atoms present in the α and β resonances were seen to decrease and increase, respectively, with increasing carbon content. This indicates a preferential occupancy from the more metallic - based on paramagnetism of conduction electrons -, presumably vacancy rich α environment to the less metallic, carbon rich β environment. Structurally, in a carbon deficient stoichiometry, the carbon atoms can be thought of as adopting arrangements around a complete or filled, close-packed Zr matrix^45,46^. Since NMR is a local spectroscopic technique, the local magnetic field induced at a carbon atom is influenced by its first and second nearest neighbours (NN). An additional effect on the shift position in metallic or semi-metallic systems; is the local density of states at the Fermi level (at carbon) i.e., a Knight shift component. The presence of vacancies may affect both factors.

**Figure 8:**
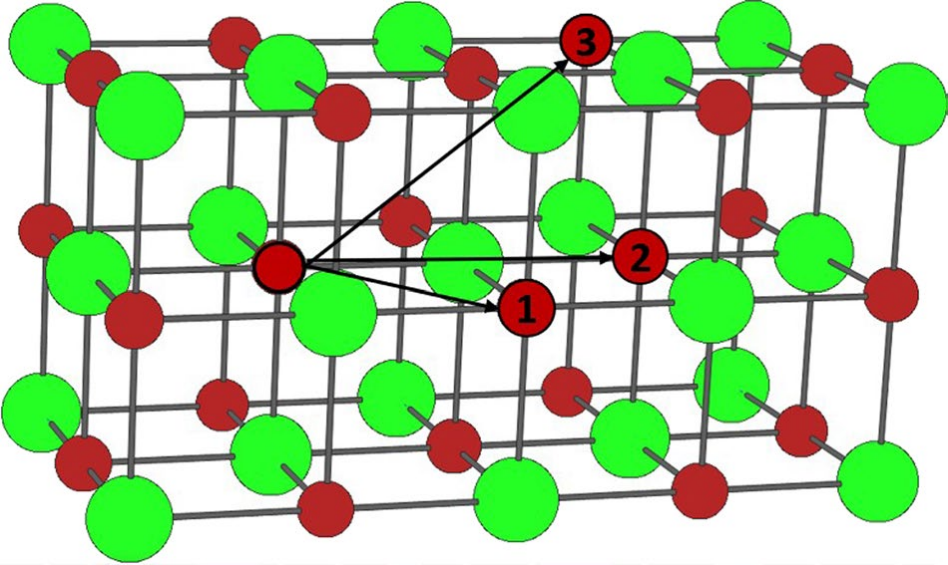
NMR sensitive carbons shown in a typical 2 × 1 cell where the carbon and zirconium atoms are identified by the green and red atoms respectively. Examples of 1^st^, 2^nd^ and 3^rd^ NN_c_ atoms are labelled by their corresponding numbers. An arrow is drawn from the central carbon atom outlined in black to each numbered NN_c_.

The inhomogeneity and systematic evolution in the sub-environment line-shapes seen in the ZrC_1*−*x_ peak of the 1500 °C sintered samples can be explored by considering vacancy ordering schemes. The NMR chemical shift is a through bond process and so near neighbours at different physical distances must be considered. Considering 108 carbon atoms in a 3 × 3 × 3 ZrC super cell, 12 carbon nearest neighbours (1NN_C_) are located at a distance of √2 d_ZrC_ from the central carbon atom, 6 carbons (2NN_C_) are located at a distance of 2d_ZrC_, and a further 24 NN_C_ are located at 3/2d_ZrC_ (Fig. 8). Considering arguments of vacancy avoidance, If all 24 3NN_c_ positions are vacated, then the resulting carbon concentration would be equivalent to ZrC_0.79_, if the next 6 2NN_C_ were vacated then the carbon concentration would be equivalent to ZrC_0.72_. The α peak was seen to emerge in the 1500 °C samples between samples with NMR corrected ZrC_0.69-0.88_. This is consistent with the major change in resonance position occurring when the sites vacated by carbons come from the closest distance at √2 d_ZrC_. Thus, the emergence of the α resonance may be attributed to the change in the removal of 2^nd^ and 3^rd^ NN_C_ to the 1^st^ NN_C_ which as result causes the line shape to shift from the β to α resonance position.

The presence of the homogeneous, unresolved, ZrC_1*−x*_ peak structure was seen in the samples sintered at 2000 °C and 1700 °C. It is expected that hot-pressing at elevated temperatures leads to higher mobility of carbon atoms, allowing them to fill a range of interstitial sites with a stochastic rather than an ordered distribution.

**Figure 9:**
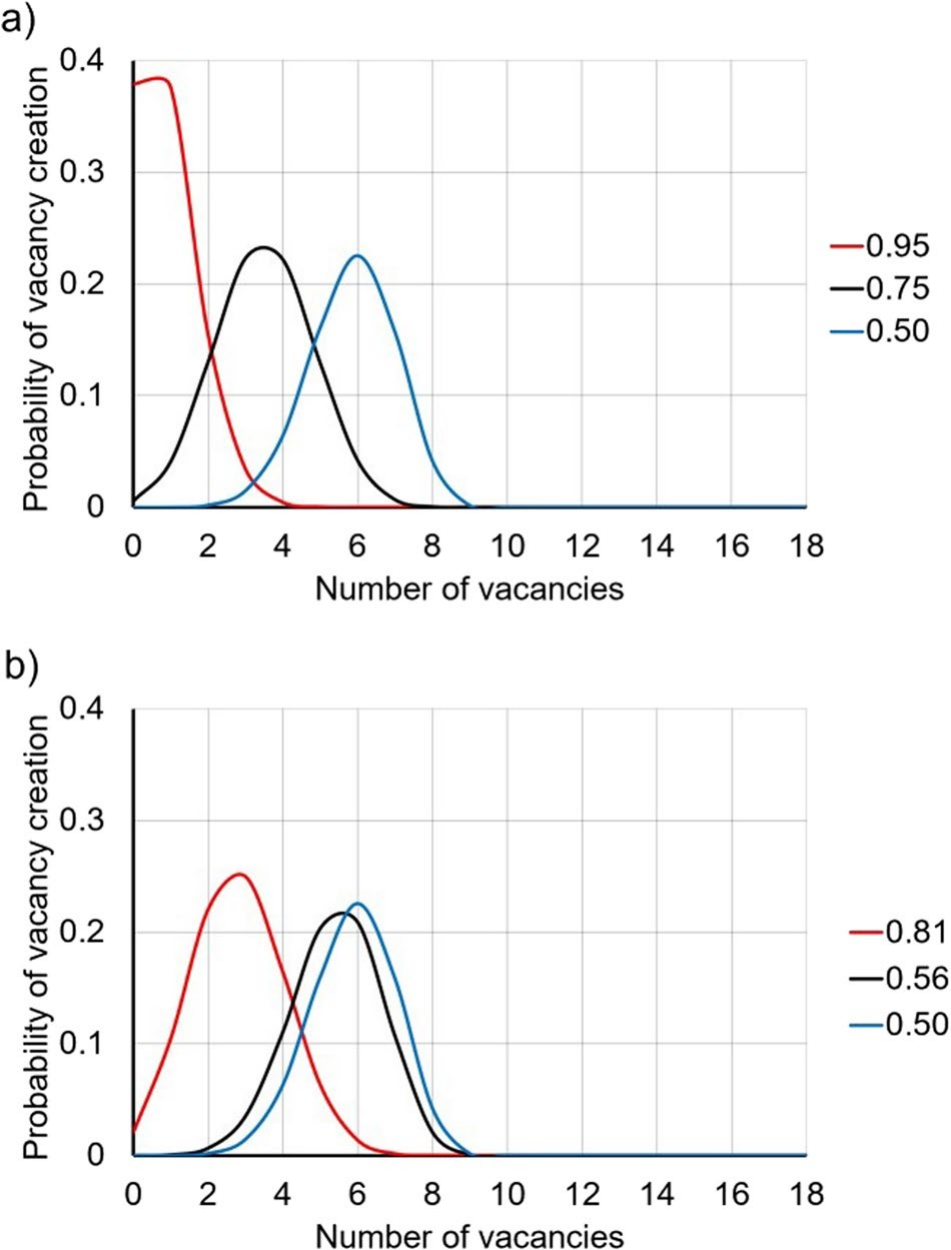
Binomial models of the vacancy distributions for NMR corrected carbon contents for (**a**) 2000 ^o^C and (**b**) 1700 ^o^C.

The binomial model can be used to determine the random distribution of vacancies over 1^st^ and 2^nd^ (Fig. 9) NN_C_ where the vacancies are statistically distributed amongst NN_C_ sites.

Figure 9 shows the binomial distribution for the carbon environments of the different schema devised. For all models it is observed that there is a high probability of obtaining a vacancy number for a given C/Zr. The binomial distributions generated show that the various samples produced by different sintering temperatures produce different probabilities of achieving different vacant sites–which could correspond to the change in the peak position observed by the 1700 °C and 2000 °C samples.

**Figure 10:**
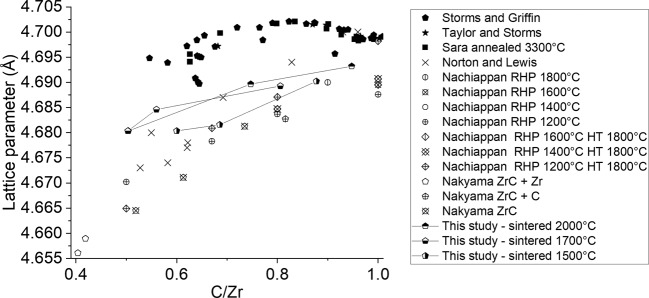
Lattice parameter from a selection of various studies^48–53^ plotted with data from this study plotted against NMR corrected C/Zr.

X-ray diffraction patterns showed no deviation from the rocksalt structure in any of the samples evidencing that the structure can remain stable for large vacancy concentrations^24,43^. This does not match the results in theoretical studies^43,44^ which imply a change in symmetry from the NaCl structure with increasing vacancy concentrations. However, it is possible microstructures may form that correspond to the determined symmetry locally but are not coherent enough for diffraction. The lattice parameters as determined by this study present lower values than have been previously shown in the literature (Fig. 10) in some cases varying by as much as 0.10 Å^47,48^.

Furthermore, earlier studies exhibit a peak in the lattice parameters value of ~4.698 Å at C/Zr ~0.83^1,2^. This peak was not observed in this study or more recent studies^48,49,53^ instead the lattice parameter appears to increase monotonically as stoichiometry is approached. This study has shown that the sintering temperature and carbon content produces a measurable impact on the lattice parameter values and their referenced stoichiometry. It is proposed that a carbon gradient and high temperature annealing can promote the ingress via diffusion of carbon into ZrC_1*−*x_ samples filling vacant sites. This increases the C/Zr of the samples resulting in incorrectly referenced lattice parameter data. The discrepancies in lattice parameter values could be due to the presence of gas interstitials contaminates^54^, existing in varying concentrations depending on fabrication techniques and substituting into vacant sites.

XRD was unable to detect the presence of excess graphite and the carbon present in the broad carbon structure. It is expected that residual carbon is dispersed within grain boundaries or the proportion is too small to be detected by XRD^55^. In addition, no other phases were discernible by XRD (see Supplementary Figure S1). This result was also consistent with other studies whose TEM analysis revealed amorphous carbon on the ~100 nanometre scale^24^. The extended network of intra and intergranular depressions as seen in the SEM images has also been observed in previous studies^12,15,19,24^. These features were seen to be comprised of sp^2^ pristine, ‘partially destroyed’ graphite and amorphous carbon structures can be seen from the Raman spectra of samples produced in this study. The pristine graphite Raman peak can be attributed to the graphite-like peak seen ~113 ppm in all ^13^C NMR spectra. As discussed previously, atoms in the broad amorphous carbon peak have a similar rapid relaxation time as graphite-like peaks but remain chemically distinct as determined from their position and line width in the NMR spectrum. Hence, the amorphous carbon and D-band present in Raman spectra can be assigned to the residual intergranular and intragranular carbon structures identified in Fig. 4 which may be remnants of soft phases that have been polished out of the sample or pores within the samples as a result of the fabrication process. As these features are not uniform in height, EDS analysis to further determine compositional details would be unreliable due to a combination of secondary emission phenomena and X-ray absorption. The presence of oxygen was not observed in the sample in XRD diffractograms or in Raman spectra^56^. However, this does not rule out their presence in the structure and further independent analysis techniques need to be undertaken to quantify the oxygen and nitrogen content of the samples.

As the samples studied via SEM were fabricated in the same hot-press run, the variation in grain size must be the result of the change in carbon content as all other conditions were the same. Assuming the decrease in grain size with increasing carbon content is the general trend for ZrC, it may explain the increase of some mechanical properties for example yield strength (via the Hall-Petch relationship^57,58^) and hardness with increasing carbon content that have been observed in the literature^1,2^.

## Conclusion

Systematic studies using ^13^C NMR have shown for the first time that quantitative carbon speciation can be obtained in reactively hot pressed and sintered ZrC_1*−x*_ samples at different temperatures. Multiple unique phases are observed by NMR in ZrC_1*−x*_ samples that are not detectable in combustion carbon analysis or observed in XRD, techniques which are commonly used to determine the C/Zr ratio. Bulk carbon analysis techniques, such as combustion carbon analysis, that are standard practice in determining the carbon content of ZrC_1*−x*_ samples are found to be inaccurate when used in isolation. This study demonstrates that bulk techniques are unable to discriminate between multiple carbon species, a proportion of which were found to be free carbon phases that were disassociated from the ZrC_1*−x*_ structure. It is recommended that the correct determination of the carbon content requires the use of bulk techniques in conjunction with local quantitative techniques such as ^13^C NMR.

The C/Zr ratio was assessed and corrected from the combustion analyser values and lattice parameters compared with previous literature values. The structure of ZrC_1*−x*_ has been shown to be stable up to 50% vacancy concentration whilst maintaining a NaCl structure which is lower than predicted by the phase diagram. The presence and systematic evolution of two unique carbon environments was observed via NMR for samples sintered at 1500 ^o^C which suggests possible systematic ordering consistent with vacancy ordering schemes calculated using Density Functional Theory.

Future work will focus on the systematic evolution of oxygen and nitrogen with sintering temperature and how long annealing times affect the values of the thermophysical properties to develop an understanding of the competing effects in interstitial site occupancy with stoichiometry and sintering temperature.

## Supplementary information

Supplementary Information.
