# Supplementary material for: An investigation of the long-range and local structure of sub-stoichiometric zirconium carbide sintered at different temperatures
Source: Sci Rep. 2020 Feb 20;10:3096. doi: 10.1038/s41598-020-59698-6 (PMC7033217; doi:10.1038/s41598-020-59698-6)
Supplement: Supplementary file 1 — Supplementary Information. [file 41598_2020_59698_MOESM1_ESM.docx]

**An investigation of the long-range and local structure of sub-stoichiometric zirconium carbide sintered at different temperatures.**


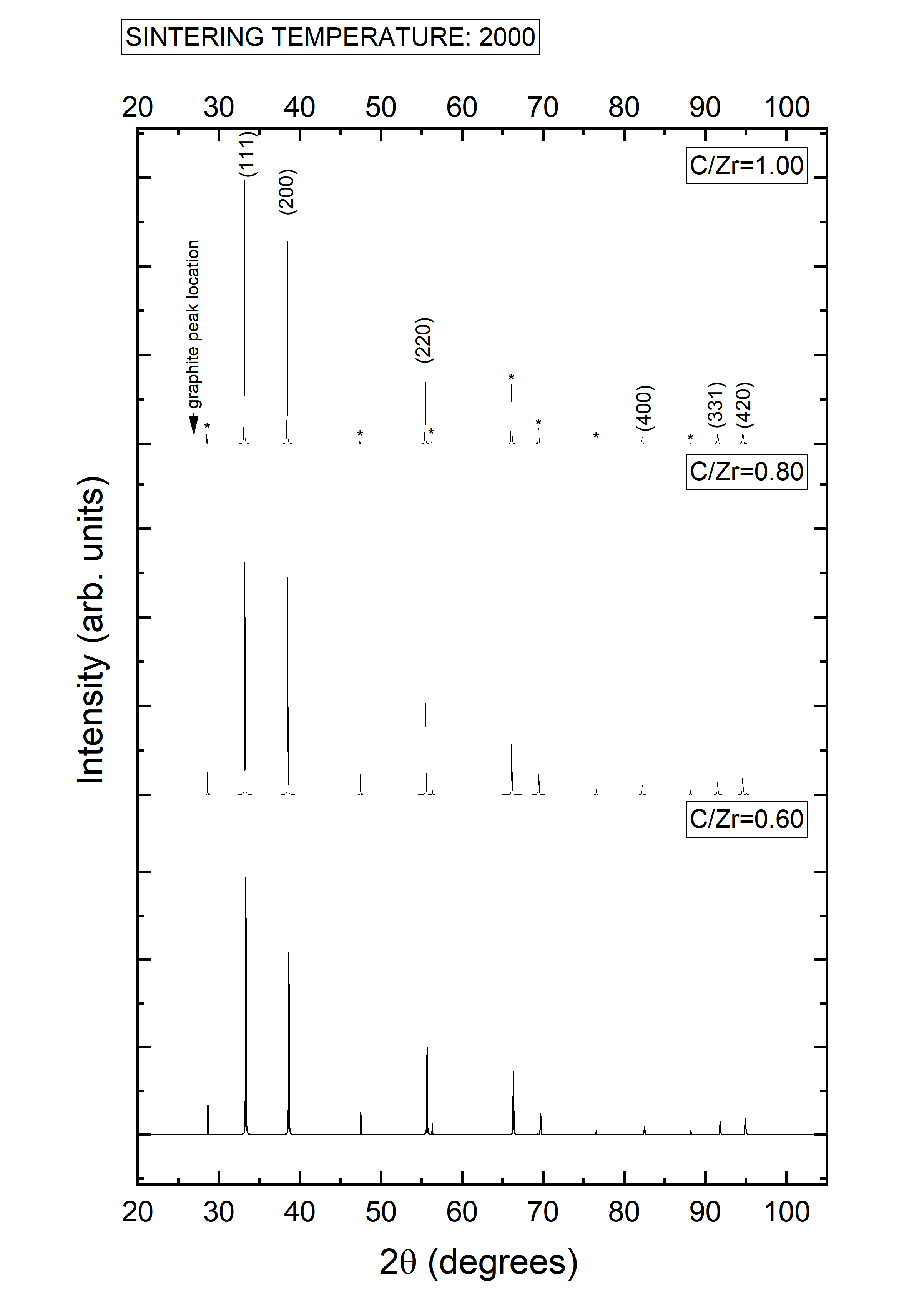
Dhan-sham B K Rana^a*^,Eugenio Zapatas Solvas^b^,William E. Lee^b,^ Ian Farnan^a^

Supplementary figure S1: XRD diffractograms of nominal stoichiometries (labelled accordingly in each graph) of samples sintered at ^2000oC^. LaB6 standard peaks are labelled with a *, ZrC peaks are indexed.

| Nominal C/Zr | ZrH_2_ (g) | C (g) | Combined mass (g) |
| --- | --- | --- | --- |
| 1.00 | 45.159 | 5.817 | 50.976 |
| 0.95 | 45.424 | 5.559 | 50.982 |
| 0.80 | 46.235 | 4.765 | 51.000 |
| 0.70 | 46.792 | 4.219 | 51.012 |
| 0.65 | 47.076 | 3.942 | 51.018 |
| 0.60 | 47.363 | 3.661 | 51.024 |

Supplementary table T1: tabulated targeted nominal C/Zr, the starting mass of the precursor powers and their corresponding combined mass.
